# P-1150. Prospective case-control study to examine clinical, biochemical, and serological indicators to predict Staphylococcal scalded skin syndrome

**DOI:** 10.1093/ofid/ofae631.1336

**Published:** 2025-01-29

**Authors:** Barnali Mitra, Debdeep Mitra

**Affiliations:** Command Hospital Air Force Bangalore, Bangalore, Karnataka, India; Assistant Professor, delhi, Delhi, India

## Abstract

**Background:**

Staphylococcal scalded skin syndrome (SSSS) typically affects infants & children, with rare occurrences in adults. There is no rapid tool or any scoring system to diagnose SSSS, where early diagnosis is the key to successful outcome.

**Methods:**

This study evaluates specific clinical & serological indicators to distinguish between maculopapular eruption & SSSS upon initial presentation. Additionally, we investigate the correlation between serum levels of thymus and activation-regulated chemokine (TARC) & the severity of SSSS & the extent of organ involvement.

In this observational study conducted prospectively, 25 patients diagnosed with SSSS & 25 control subjects with maculopapular rash were enrolled.

**Results:**

The diagnostic efficacy of various factors, including TARC level ( >612.25 pg/ml), total body surface area (TBSA >30%), hsCRP ( >5mg/l), leucocytosis ( >12X 10^9^ cells/L), & aspartate transaminase ( >90 U/L), showed statistical similarity to the diagnosis of SSSS. While baseline serum TARC levels proved to be a reliable screening tool, they did not predict the severity or outcome of SSSS. However neutrophil extracellular traps (NETs) level did correlate positively with disease severity & duration of hospital stay.

Through multivariable regression analysis, we have formulated a novel model that integrates TBSA at baseline, neutrophil percentage in leucocytosis, & hsCRP, surpassing the predictive capacity of these parameters when considered individually. The resulting equation with the highest odds for diagnosing SSSS is as follows:

Odds of SSSS (present) = Antilog [-11.26 + 0.3 x Total BSA + 0.30 x hsCRP – 0.1 x neutrophil (%)].

This composite model (incorporating TBSA at baseline, neutrophil percentage, and hsCRP) achieved a sensitivity of 96% and specificity of 100% at a cutoff point of 7.8.

**Conclusion:**

The equation developed from our research is straightforward to apply and suitable for use in settings with limited resources. It facilitates the identification of patients who are more likely to have SSSS, enabling the early initiation of definitive treatment. Likewise, it helps in minimizing unnecessary extensive investigations for patients unlikely to have SSSS. Neutrophil extracellular traps (NET) can be definitely used as a prognostic marker in patients with severe SSSS.

**Disclosures:**

**All Authors**: No reported disclosures

